# First Trimester Spontaneous Uterine Rupture in a Young Woman with Uterine Anomaly

**DOI:** 10.1155/2014/967386

**Published:** 2014-01-16

**Authors:** Esra Nur Tola

**Affiliations:** Department of Obstetrics and Gynecology, Faculty of Medicine, Suleyman Demirel University, Isparta, Turkey

## Abstract

Spontaneous uterine rupture is a life-threatening obstetrical emergency carrying a high risk for the mother and the fetus. Spontaneous uterine rupture in early pregnancy is very rare complication and it occurs usually in scarred uterus. Uterine anomalies are one of the reasons for spontaneous unscarred uterine rupture in early pregnancy. Obstetricians must consider this diagnosis when a pregnant patient presented with acute abdomen in early pregnancy. We present a case of spontaneous uterine rupture at 12 weeks of gestation in 24-year-old multigravida who had uterine anomaly presenting as an acute abdomen. Our preoperative diagnosis was ectopic pregnancy. Emergency laparotomy confirmed a spontaneous uterine rupture. Uterine anomaly is a risk factor for spontaneous uterine rupture in the early pregnancy. Clinical signs of uterine rupture in early pregnancy are nonspecific and must be distinguished from acute abdominal emergencies.

## 1. Introduction

Rupture of a pregnant uterus is one of the life-threatening complications associated with obstetric practice [1]. There are several risk factors associated with uterine rupture (UR), but the most common is a previous Cesarean section. Unscarred uterine rupture (UUR) is a rare event that usually occurs in late pregnancy or during labour. Risk factors for UUR include high parity, placental abnormalities, and uterine anomaly. UUR during pregnancy, especially before the end of the second trimester, occurs relatively rarely and is associated with high mortality and morbidity for both the fetus and mother.

We here report a case of a spontaneous unscarred uterine rupture (SUUR) in early pregnancy, in a woman with a bicornuate uterus.

## 2. Case Report 

**Figure 1:**
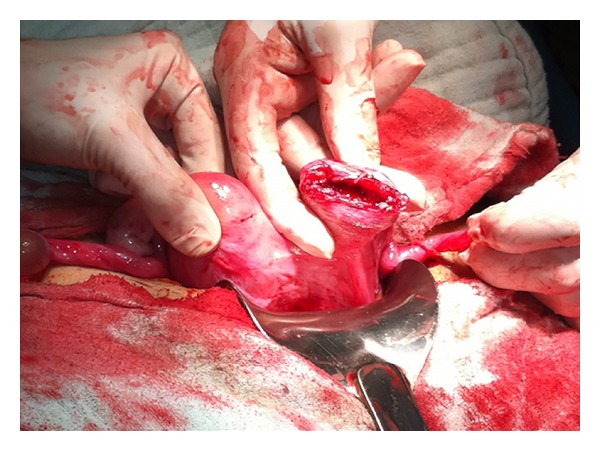
Fundal uterine rupture in the left part of the bicornuate uterus.

A 24-year-old woman was admitted to our department with 3-month amenorrhea and sudden, severe, generalized abdominal pain and vaginal bleeding of 2-hour duration. Her first pregnancy resulted in abortus at 8 gestational weeks, but no surgical procedure was performed. On physical examination, the patient was pale with a pulse rate of 130 beats per minute (bpm) and a blood pressure of 80/40 mmHg. The abdomen was tender. Ultrasound examination revealed a 12-week consistent, fetal heartbeat negative pregnancy in the left tuboovarian area and free fluid in the Douglas pouch. Haemoglobin concentration was 7 g/dL. Due to unstable vital signs, two units of screened blood were cross-matched, and the patient was rushed to the operating room. Our preoperative diagnosis was ectopic pregnancy. Laparotomy revealed haemoperitoneum of 1 liter. Further inspection showed a bicornuate uterus and a 2 cm fundal uterine rupture in the left part of the uterus (Figure 1). In the abdomen, a 12-week pregnancy consistent fetus was found. The products of conception were removed, and uterine repair was performed with a size of 1 vicryl suture. The patient's postoperative recovery was uneventful and she was discharged on her third postoperative day. The patient was counselled on the need to be delivered by elective Cesarean section in subsequent pregnancies.

## 3. Discussion

UUR is a rare, life-threatening complication during pregnancy, with an incidence rate of 1 : 17,000–20,000 [2] and a mortality and morbidity rate estimated to be between 20.8% and 64.6% [3].

UR is usually observed in association with uterine scarring either in late pregnancy or during labour [4]. First and early second trimester UURs are very rare, and there are only a few cases in literature describing first and early second trimester UURs [4–7]. In our case, UUR occurred in the twelfth week of pregnancy.

Although a prior Cesarean section is a major risk factor for UR, in patients with unscarred uteri, high parity (≥4 births) is a major risk factor. Other risk factors for UUR include abnormal placentation, uterine anomalies, obstetric manoeuvres, malpresentations, excessive uterine expressions, curettage, injudicious use of oxytocin, uterine diverticula [8], and chronic corticosteroid use [3], whereas some cases have no obvious cause [9]. In our case, uterine anomaly may be implicated in the UR, because the patient had a bicornuate uterus, and there were no other obvious risk factors. Singh and Jain and Kahyaoglu et al. previously reported UUR with uterine anomaly in the first trimester [6, 10]. A few cases of UUR in the early trimester with no previous risk factors [5, 7] and as a result of placenta percreta, have been reported [11, 12].

Clinical signs of UR in early pregnancy are nonspecific and must be distinguished from acute abdominal emergencies. Abdominal pain, vaginal bleeding, and vomiting are classic findings. Differential diagnoses are bleeding corpus luteum, heterotropic or ectopic pregnancy, and molar pregnancy with secondary invasion [4]. The most relevant differential diagnosis is ectopic pregnancy [6]. Sometimes ultrasound has limited value and urgent surgery is necessary to prevent catastrophic sequelae. An emergency laparoscopy or laparotomy is needed for the correct diagnosis and to enable the necessary treatment to take place. Early correct diagnosis and proper management are necessary to decrease the high maternal and fetal mortality and morbidity rates associated with UR. In our case, our initial diagnosis was ectopic pregnancy, and we performed emergency laparotomy after judging that laparoscopic instrumentation was deficient and because of the unstable vital signs of the patient.

UUR usually occurs in the lower segment (the weakest part) of uterus [13]. If the rupture part is the fundus, as in our case, the diagnosis is often delayed because the haemorrhage is not revealed immediately, as blood collects in the intraperitoneal space [13].

Early surgical intervention is usually the key to successful treatment of UR. Treatment will primarily depend on the extent of the lesion, the parity, age and condition of the patient, and expertise of the surgeon. Although in the past hysterectomy has been suggested for the therapeutic management, recent studies have shown that the suture can be performed with bilateral tubal ligation or suture without tubal ligation in women who wish to preserve fertility with a recurrence risk of UR assessed to be between 4 and 19% at a subsequent pregnancy [14]. For this reason the patient must be counselled on the need to undergo a Cesarean section in all future pregnancies. In our case we performed uterine suture without tubal ligation because our patient had no previous children.

## 4. Conclusion 

In conclusion, UUR in early pregnancy is a rare and potentially catastrophic event. Uterine anomalies are one of the reasons of UUR. The current case highlights uterine anomaly as a risk factor for spontaneous UR in the first trimester of pregnancy. Clinical signs of UR in early pregnancy are nonspecific and must be distinguished from other acute abdominal emergencies.

## Abbreviations

UUR: Unscarred uterine rupture
SUUR: Spontaneous unscarred uterine rupture
UR: Uterine rupture.
